# Co-Mutations and Possible Variation Tendency of the Spike RBD and Membrane Protein in SARS-CoV-2 by Machine Learning

**DOI:** 10.3390/ijms25094662

**Published:** 2024-04-25

**Authors:** Qiushi Ye, He Wang, Fanding Xu, Sijia Zhang, Shengli Zhang, Zhiwei Yang, Lei Zhang

**Affiliations:** 1 MOE Key Laboratory for Nonequilibrium Synthesis and Modulation of Condensed Matter, School of Physics, Xi’an Jiaotong University, Xi’an 710049, China; yeqiushi@stu.xjtu.edu.cn (Q.Y.);; 2 School of Life Science and Technology, Xi’an Jiaotong University, Xi’an 710049, China

**Keywords:** SARS-CoV-2, co-mutations, mutational synergy, sequence-to-sequence transformer model, sequence analysis

## Abstract

Since the onset of the coronavirus disease 2019 (COVID-19) pandemic, SARS-CoV-2 variants capable of breakthrough infections have attracted global attention. These variants have significant mutations in the receptor-binding domain (RBD) of the spike protein and the membrane (M) protein, which may imply an enhanced ability to evade immune responses. In this study, an examination of co-mutations within the spike RBD and their potential correlation with mutations in the M protein was conducted. The EVmutation method was utilized to analyze the distribution of the mutations to elucidate the relationship between the mutations in the spike RBD and the alterations in the M protein. Additionally, the Sequence-to-Sequence Transformer Model (S2STM) was employed to establish mapping between the amino acid sequences of the spike RBD and M proteins, offering a novel and efficient approach for streamlined sequence analysis and the exploration of their interrelationship. Certain mutations in the spike RBD, G339D-S373P-S375F and Q493R-Q498R-Y505, are associated with a heightened propensity for inducing mutations at specific sites within the M protein, especially sites 3 and 19/63. These results shed light on the concept of mutational synergy between the spike RBD and M proteins, illuminating a potential mechanism that could be driving the evolution of SARS-CoV-2.

## 1. Introduction

Since the initial case, identified in December 2019, the coronavirus disease 2019 (COVID-19) pandemic caused by severe acute respiratory syndrome coronavirus-2 (SARS-CoV-2) [1,2,3] has garnered worldwide attention [4]. As of 12 June 2023, the World Health Organization (WHO) reported a remarkable 767 million COVID-19 cases worldwide, with 6.9 million deaths (https://covid19.who.int/ (accessed on 12 June 2023)). Despite the widespread distribution of vaccines, the rapid mutation rate of SARS-CoV-2 poses ongoing challenges to immune responses and vaccine efficacy [5]. Consequently, there is an urgent need for continuous viral surveillance, the creation of innovative vaccines, and the meticulous testing of vaccination strategies to effectively confront the ongoing threat of COVID-19 [6,7,8,9,10].

### 1.1. SARS-CoV-2 Structural Proteins

**Figure 1:**
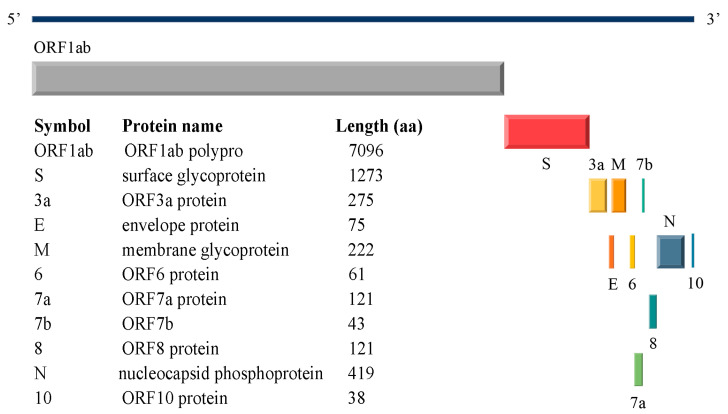
SARS-CoV-2 reference genome (NC_045512.2).

SARS-CoV-2 virus comprises four structural proteins [11]: the spike (S), membrane (M), nucleocapsid (N), and envelope (E) proteins. Figure 1 provides detailed insights into the SARS-CoV-2 reference genome (NC_045512.2) [12]. SARS-CoV-2 is an enveloped virus that uses membrane fusion [13,14] to enter host cells [15,16]. The successful infection cycle of SARS-CoV-2 relies heavily on its structural proteins, especially the S and M proteins. The S protein is assembled into a homotrimer structure and is crucial for viral entry by recognizing host cell receptors and mediating membrane fusion [17,18]. A particularly significant component of the S protein is the receptor-binding domain (RBD), which directly interacts with host receptors [19]. The M protein plays a role in the assembly of virions and the process of membrane budding [20,21], while the N protein facilitates the transcription and replication of viral RNA within the host cells [22,23]. The E protein forms a cation channel that is vital for the pathogenicity of the virus [24].

Like SARS-CoV-2, the Influenza A virus (IAV), another enveloped virus, triggers disease by leveraging a pair of complementary proteins: [25]: hemagglutinin (HA) [26], which mediates viral entry, and neuraminidase (NA) [27], which facilitates viral egress [28,29]. The functional antagonism exhibited by the HA and NA proteins of IAV offers a valuable reference for the evolutionary mechanisms of SARS-CoV-2, such as the functional linkages between the S and M proteins.

### 1.2. SARS-CoV-2 Variants

SARS-CoV-2 is a novel evolutionarily divergent RNA virus [30]. Unlike DNA viruses, RNA viruses exhibit higher error rates during replication and possess less efficient error-correcting mechanisms [31]. Consequently, it leads to a dramatically high mutation and evolution rate, which is correlated with virulence modulation and evolvability [32,33,34]. SARS-CoV-2 has a spontaneous mutation rate of approximately 1.3 × 10^−6^ ± 0.2 × 10^−6^ per base per infection cycle, based on the accumulation of mutation frequencies and excluding genes under selective pressure [35]. The emergence of these viral variants has led to more contagious strains and instances of vaccine breakthrough infections [36]. Among the four structural proteins, the S protein, particularly its RBD, is the primary focus of current research due to its role in immune evasion [37,38,39]. In contrast, the amino acid sequences of the M, N, and E proteins exhibit greater stability and conservation.

The World Health Organization (WHO) classified some SARS-CoV-2 variants as variants of concern (VOC), including Alpha (B.1.1.7), Beta (B.1.351), Gamma (P.1), Delta (B.1617.2), and Omicron (B.1.1.529). The Omicron variant, characterized by its high transmissibility and prevalence of asymptomatic cases, has become the dominant strain in many countries [40,41,42]. It was found that the extensive spike RBD mutations in Omicron allowed it to evade immune responses targeted at the original strain [43]. The WHO has reported novel M protein mutations in the Omicron variant, such as D3G/N, Q19E, and A63T (https://www.who.int/activities/tracking-SARS-CoV-2-variants (accessed on 12 June 2023)). With the Omicron variants, we have observed a synchronous increase in mutations in the spike RBD and M proteins, which is a phenomenon not previously seen. Based on its structure and function, the spike protein (especially the RBD) mediates viral entry, replication, and the assembly of new virions [16], and the M protein facilitates the release from host cells. We hypothesized that the spike RBD and M proteins have a mutational synergy tendency which may enhance the viral infectivity and the capacity to avoid host immune responses.

Interestingly, the HA and NA proteins of IAV not only share functional parallels with the spike RBD and M proteins of SARS-CoV-2 but also have exhibited a pattern of co-mutation synergy. IAV undergoes antigenic drift, leading to a multitude of variants that can evade the host immune system [44,45]. This trait has contributed to its high transmissibility and the occurrence of four global pandemics (https://www.cdc.gov/flu/pandemic-resources/ (accessed on 12 June 2023)). The synergistic genomic interactions between the HA and NA proteins are a major force underlying IAV’s evolution [46,47,48]. The mutational synergy between HA and NA at the sequence level is the potential restrictive factor for IAV’s evolution [49,50]. Drawing from research on the mutational synergy of the HA and NA proteins, we try to explore the possible variation tendency of the spike RBD and M proteins from an evolutionary perspective.

### 1.3. Mutational Correlations Analysis

Regarding mutation analysis, Göbel et al. were considered the pioneers in calculating the mutational correlations between different positions using the Pearson correlation coefficient [51]. However, the advent of machine learning has redirected the focus towards addressing the exploration of contact interactions between protein sequence positions as a pattern recognition challenge, leading to notable enhancements in predictive accuracy. In 2015, Figliuzzi et al. introduced a mutation effect prediction method based on mean field Direct Coupling Analysis (DCA), which predicts the phenotypic effects of mutation sequences relative to the wild-type sequence by statistically scoring each variant sequence [52]. This approach demonstrated a higher accuracy compared to models that independently analyze individual positions. Particularly, Hopf et al. developed EVmutation [53] based on Pairwise Likelihood of Mutation Directed Coupling Analysis (PLMDCA) in 2017, which deduces the mutational phenotype by emulating the interactions among all protein residues and concurrently evaluates the influence of mutations.

While previous studies were predominantly concentrated on individual proteins, there has been a scarcity of comprehensive studies examining the co-mutation tendencies between pairs of proteins. To bridge this research gap, an attention-based neural network model, termed the Sequence-to-Sequence Transformer Model (S2STM), has been introduced [54], which has effectively facilitated mutual mapping between the HA and NA sequences of IAV. In this context, the S2STM is utilized to delve into the co-mutation tendencies of the spike RBD and M proteins. The S2STM incorporates a multi-head attention mechanism that enhances global context for information processing, mitigating overfitting and enhancing interpretability. Moreover, the computational complexity of the S2STM is invariant to the distance between positions, which is a distinct advantage over convolutional neural networks (CNNs) when dealing with longer sequences. Compared to recurrent neural network (RNN) models, the S2STM significantly boosts computational efficiency through the capability for efficient parallel processing. Consequently, the S2STM is particularly adept at managing high-throughput sequence data and at uncovering potential mutation trends between the spike RBD and M proteins.

In this study, our attention is directed towards examining the co-mutations and potential evolutionary patterns between the spike RBD and M proteins at the amino acid level, utilizing the S2STM method. By analyzing the mutation distribution of the S and M proteins and employing the EVmutation to investigate the association between the spike RBD mutations, we tentatively concluded that three co-mutations (G339D-S373P-S375F, S371F/L-S373P-S375F, and Q493R-Q498R-Y505H) in the spike RBD sequence were likely to induce mutations at sites 3/19/63 in the amino acid sequences of the M protein. After initially revealing a strong correlation between the co-mutations, we further tested the correlations based on the S2STM. Finally, we concluded that the co-mutations G339D-S373P-S375F and Q493R-Q498R-Y505 had the highest probability of inducing mutations at sites 3 and 19/63 in the amino acid sequence of the M protein. Our study serves as a bridge between natural language processing and viral evolution, shedding light on the associations between mutations in the spike RBD and M proteins at the sequence level.

## 2. Results

Section 2.1 shows the results of the mutation analysis. Section 2.2 shows the possible variation tendency of the spike RBD and M mutations by analyzing the sequence translation predicted by the S2STM.

### 2.1. Variation Analyses at the Amino Acid Level

#### 2.1.1. Results of Single Mutation Analysis

Deletions, replacements, and insertions are primary natural phenomena in viral evolution [45]. We analyzed the distribution of amino acids in the spike RBD and M sequences from NCBI (https://www.ncbi.nlm.nih.gov/data-hub/taxonomy/2697049/ (accessed on 20 April 2022)) (Supplementary Table S1). Supplementary Table S1 indicates that the M sequences were relatively conserved, and only four sites exhibited mutations: D3G, Q19E, A63T, and I82T. In contrast, the mutations in the spike RBD were more complex and varied. Combining the mutation information published by the WHO (Table 1), we found that both the M sequences and the spike RBD sequences exhibited a large number of new mutations in the Omicron variant. To explore mutational synergy, we focused on exploring these significant mutations. In this work, we analyze a total of 39,847 sequences.

**Figure 2:**
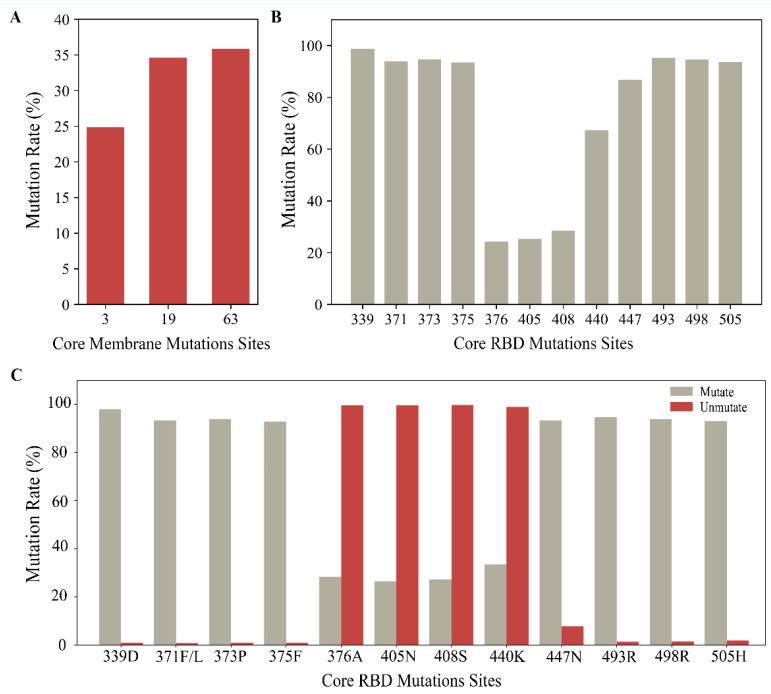
The mutation rates in core M and RBD mutations. (**A**) The mutation rates in core M mutations. (**B**) The mutation rates in core RBD mutations. (**C**) The mutation rates of the core RBD mutations when any or none of sites 3, 19, and 63 in the M sequences were mutated. The light gray shows the mutation rate of core RBD mutations when any of the sites 3, 19, and 63 in the M sequences were mutated (3G, 19E, and 63T). The dark red color shows the core RBD mutation rate when sites 3, 19, and 63 in the M sequences were unmutated (D3, Q19, and A63).

The core mutations in the M protein are D3G, Q19E, and A63T. The core mutations in the spike RBD include G339D, S371F/L, S373P, S375F, T376A, D405N, R408S, N440K, S447N, Q493R, Q498R, and Y505H. The mutation rates for these are depicted in Figure 2A,B.

Additionally, we calculated the mutation rate of the core RBD mutations when any or none of the sites 3, 19, and 63 in the M sequences were mutated, as illustrated in Figure 2C. Notably, eight core RBD mutations (G339D, S371F/L, S373P, S375F, S447N, Q493R, Q498R, Y505H) occurred concurrently with mutations in the M sequences (the probability was 90% for all the mutations). It tentatively indicated a correlation between certain mutations in the M and spike RBD sequences.

#### 2.1.2. Results of Multiple Mutation Analysis

**Figure 3:**
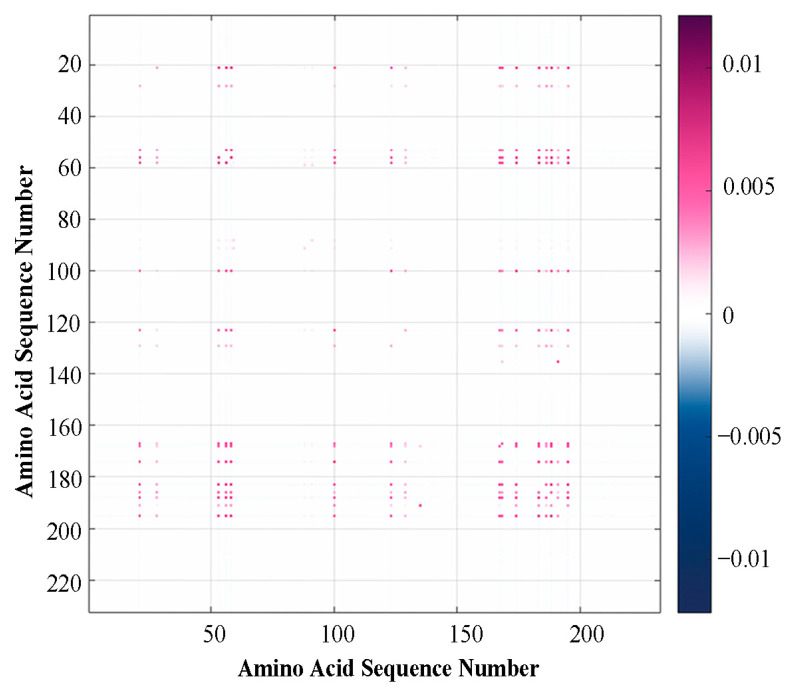
The coupling intensity map in the spike RBD using EVmutation.

Given the correlation between the amino acid mutations, we utilized EVmutation to analyze the mutation profile of the spike RBD and generated a coupling strength map, as depicted in Figure 3. The coupling strength map represents the strength of the evolutionary couplings between the amino acid positions in amino acid sequences. It appears that some amino acids exhibit the co-evolutionary relationships from Figure 3, which implies the significance of conducting further research.

**Figure 4:**
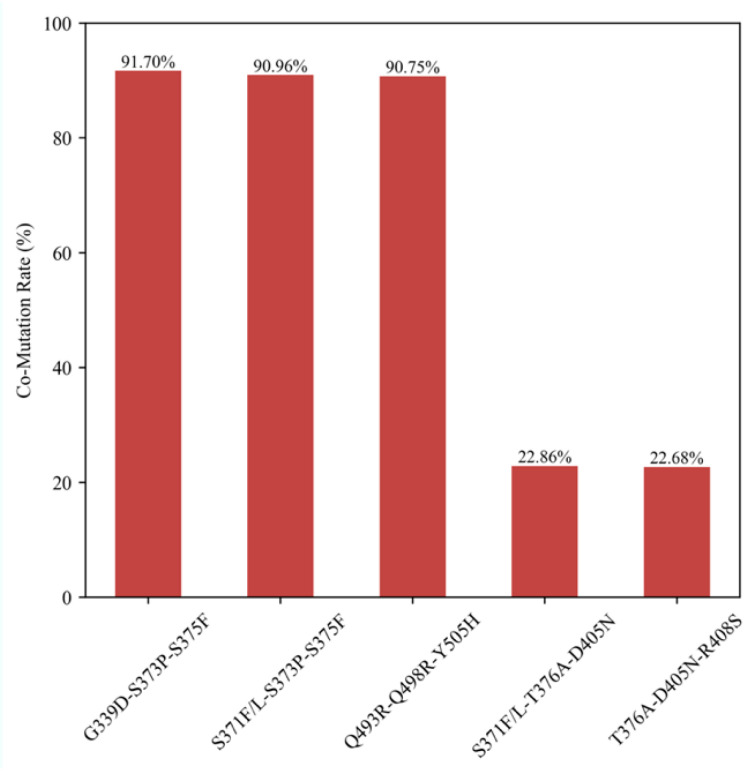
The co-mutation rate of core RBD mutations when any of the sites 3/19/63 in the M sequences were mutated.

After successive filtering based on the scores and the co-occurrence of mutations, we identified that certain core RBD mutations exhibited strong correlations. These included G339D-S373P-S375F, S371F/L-S373P-S375F, Q493R-Q498R-Y505H, S371F/L-T376A-D405N, and T376A-D405N-R408S. Combined with analyzing their co-mutation rates when any of the sites 3/19/63 in the M sequences were mutated (Figure 4), we preliminarily concluded that the RBD mutations G339D-S373P-S375F, S371F/L-S373P-S375F, and Q493R-Q498R-Y505H were likely to induce mutations at sites 3/19/63 in the M sequences.

### 2.2. Possible Variation Tendency Identified by S2STM

**Figure 5:**
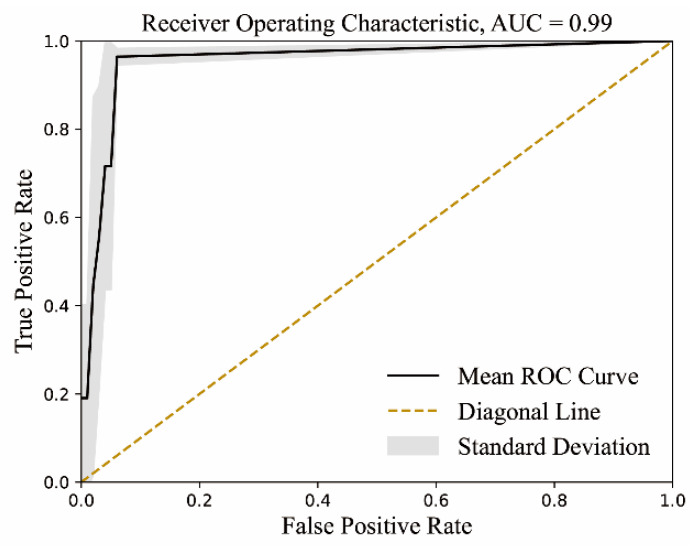
The Receiver Operating Characteristic Curve in testing datasets.

The S2STM was trained and evaluated with the spike RBD sequences and the M sequences. In the testing datasets for the M protein, the model achieved an accuracy of 99%. To assess the model’s performance, we conducted calculations of the Pearson correlation coefficient (PCC) [55] and Hamming distance [56], respectively, in the embedding space and primitive space. The mean correlation coefficient for the testing datasets was 0.964, indicating a strong correlation (>0.8). The variance was found to be 1.475 × 10^−4^. The Hamming distance, frequently utilized to measure the similarity between two strings, assigns a distance value of 0 to indicate exact similarity. In our test datasets, 85.3% of the distance values were below 5, with 47.8% of them being 0. Moreover, the testing datasets exhibited a statistically significant and strong Area Under the Curve (AUC) [57] of 0.99, as demonstrated by the Receiver Operating Characteristic Curves (ROCs) presented in Figure 5. These findings underscore the robustness and accuracy of the S2STM model, which effectively learns and establishes the mapping relationship between the RBD and M sequences. Consequently, the model can be employed to assess and determine the association of site-specific mutations between the RBD and M sequences.

**Figure 6:**
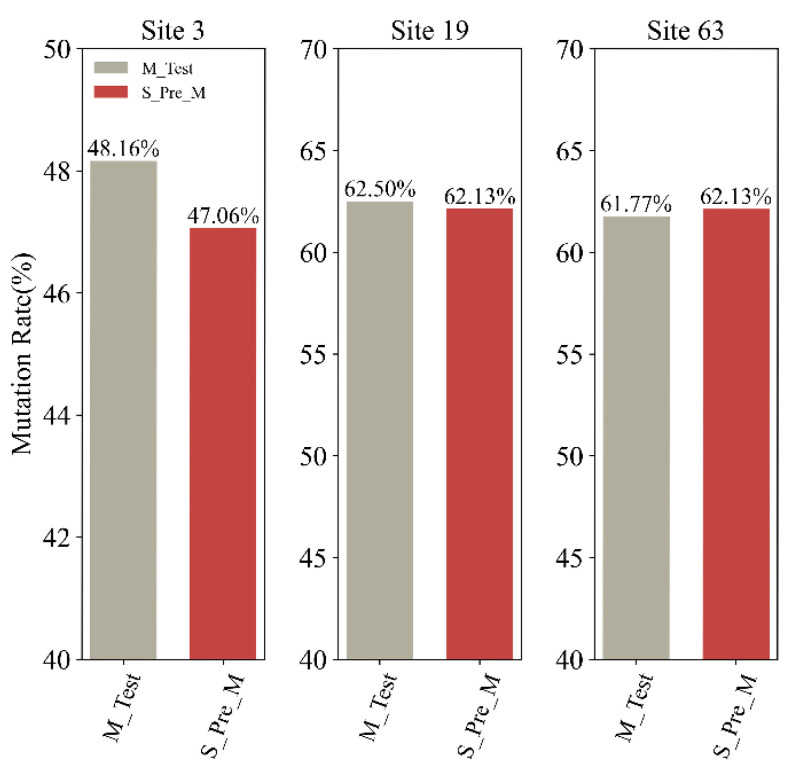
The difference in the mutation rate of the core sites in the M sequences between the baseline data (“M_Test”) and “S_Pre_M”. The light gray shows the mutation rate in “M_Test”, and the dark red shows the mutation rate in “S_Pre_M”. The detailed mutation rates of sites 3/19/63 are shown from left to right.

To further analyze the co-mutations, we utilized the S2STM to translate the revised testing datasets, as outlined in the Section 4. This process yielded the translated M sequences, which were designated as “S_Pre_M”, “S_G339-S373-S375_Pre_M”, “S_339D-373P-375F_Pre_M”, “S_S371-S373-S375_Pre_M”, “S_371F/L-373P-375F_Pre_M”, “S_Q493-Q498-Y505_Pre_M”, and “S_493R-498R-505H_Pre_M”. Additionally, we compared the mutation rates between the baseline data (“M_Test”) and “S_Pre_M” to validate the accuracy. The discrepancies were found to be no greater than 1.10% (with specific values being 1.10%, 0.37%, and 0.36%), as depicted in Figure 6. Negligible numerical deviations underscore the accuracy of the model.

**Figure 7:**
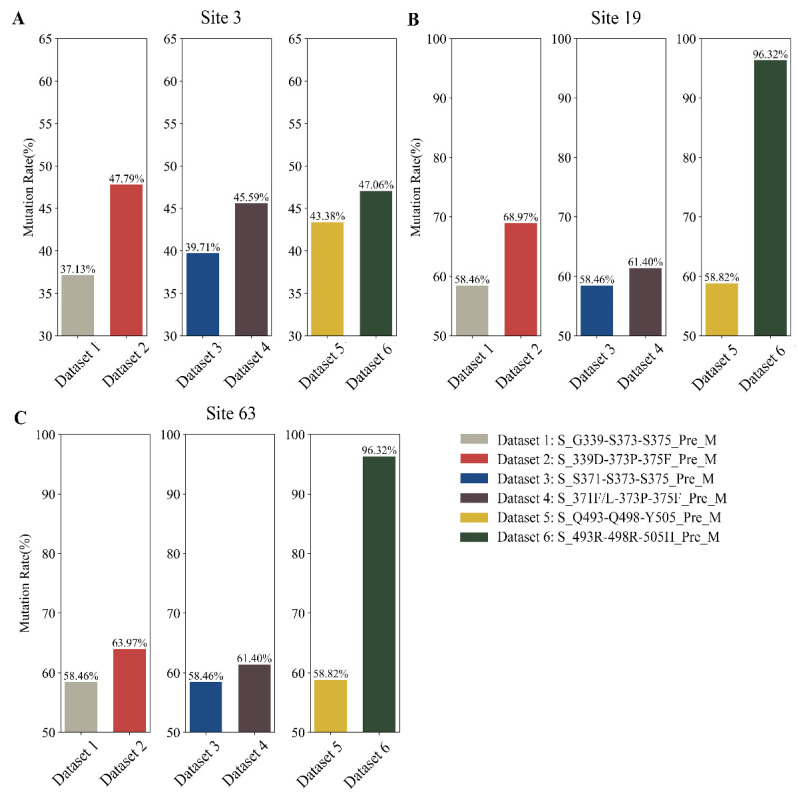
The mutation rate of the core sites (3/19/63) in the translated M sequences. Light gray, dark red, dark blue, dark gray, yellow, and dark green indicate the translation results in “S_G339-S373-S375_Pre_M”, “S_339D-373P-375F_Pre_M”, “S_S371-S373-S375_Pre_M”, “S_371F/L-373P-375F_Pre_M”, “S_Q493-Q498-Y505_Pre_M”, and “S_493R-498R-505H_Pre_M”, respectively. (**A**) The mutation rate at site 3 in the M sequences. (**B**) The mutation rate at site 19 in the M sequences. (**C**) The mutation rate at site 63 in the M sequences.

Next, we computed the mutation rates for the core sites (3/19/63) in the M sequences (Figure 7). Our analysis revealed that following the mutation of the core co-mutations in the RBD, the incidence of mutations at site 3 in the M sequences rose by 3.68%, 5.88%, and 10.66%; mutations at site 19 increased by 37.50%, 2.94%, and 10.51%; and mutations at site 63 increased by 37.50%, 2.94%, and 5.51%, respectively. Among them, the co-mutations G339D-S373P-S375F had the highest probability of inducing mutations at site 3 in the M sequences, and the co-mutations Q493R-Q498R-Y505 had the highest probability of inducing mutations at sites 19 and 63 in the M sequences. Given these analogous outcomes, we hypothesized that sites 3 and 19 exhibit synergistic mutational relationships. Through sequence alignment and variation analysis, we established the relationship between the RBD and M sequences at the amino acid level.

## 3. Discussion

Based on the functions of the S RBD and M proteins and drawing from research on the mutational synergy of the HA and NA proteins, we try to explore the possible variation tendency of the spike RBD and M proteins from an evolutionary perspective. Until late 2022, the predominant COVID-19 vaccines were formulated based on the S antigen of early viral variants. However, the emergence of the Omicron variant has resulted in an increased incidence of mutations within the spike RBD, which has posed significant challenges in the development of universal vaccines. The spike RBD, which plays a crucial role in viral entry and immune evasion, has become a major focus for vaccine development efforts [19]. The mutations that have been identified have been a popular topic of discussion, and the RBD mutations, in particular, have been extensively studied.

The mutations Q493R, S371L, S373P, and S375F have been reported to enhance binding to the ACE2 receptor [58]. Additionally, the mutations S371L, S375F/L, Q493R, and Q498R are suggested to potentially introduce spatial steric hindrance or disrupt specific hydrogen bonds [40]. The M amino acid sequences are highly conserved, with only a few atypical mutations observed in the Omicron variant. The effects of M protein mutations have not been extensively studied, with limited research conducted to date. As a distinct variant, the Omicron variant has been identified as the predominant strain of SARS-CoV-2 since December 2021. Compared to earlier strains, the Omicron variant is less symptomatic, less lethal, and has a shorter recovery time, but it spreads rapidly through the population [59].

Notably, the co-mutations identified in the spike RBD are also present in nearly all the Omicron sublineages. Despite a comprehensive investigation, no relevant biochemical experiments have been found to confirm the synergistic effects of mutations between the spike RBD and M proteins. Consequently, experimental efforts are expected in order to validate the findings regarding mutational synergy in the near future.

However, the mutation synergy between the spike RBD and M proteins identified through our model still can hold meaningful implications for therapeutic strategies and vaccine development [60,61]. For instance, vaccines could potentially be designed to target key residues involved in the synergistic interaction. Such a design might elicit immune responses that disrupt viral entry or replication, thereby inhibiting the spread of the virus and potentially conferring broader protection against emerging viral variants. Understanding the potential interactions between the mutations within these proteins provides a novel perspective on the plausible evolutionary trajectory of the virus and its infectivity, thereby facilitating the development of vaccines that are more effective against a range of viral strains.

The XBB variant [62,63,64] has been detected in 35 countries and has gained a worldwide presence, with a global prevalence of 1.3%. It is a recombinant Omicron subtype resulting from the BA.2.10.1 and BA.2.75 sublineages. Although the sequences of the XBB variant are not included in our dataset, they serve as a valuable validation set for assessing the performance of our model. Core mutations in the spike RBD (G339H, S371F, S373P, S375F, Q498R, and Y505H) have been identified in the XBB variant, based on the available amino acid sequences (https://covdb.stanford.edu/variants/omicron_ba_2/ (accessed on 16 April 2024)). It is notable that site 339 was mutated from G to H in the XBB. According to our conclusions, G339D-S373P-S375F is highly likely to induce mutations at site 3 in the M sequences. However, with a different mutation observed at site 339 in the RBD sequences, site 3 in the M sequences remains unmutated (D3). This suggests that the potential variation tendencies of the spike RBD and M proteins may also be relevant to the XBB variant, highlighting the broad applicability of this study.

## 4. Materials and Methods

Here, we describe the methods of sequence retrieval and preparation, mutation synergy analysis using EVmutation, and sequence translation based on S2STM in detail: (1) sequence preparation: preliminary deletion and alignment of the S and M amino acid sequences; (2) mutational synergy analysis: analysis of the mutation distribution of the filtered S and M amino acid sequences and use of EVmutation to explore the association between mutations in the spike RBD; and (3) sequence translation: testing the correlations of mutations based on S2STM.

### 4.1. Sequence Retrieval and Preparation

Complete or near-complete amino acid sequences of the S and M proteins from SARS-CoV-2 viruses were retrieved from NCBI (https://www.ncbi.nlm.nih.gov/data-hub/taxonomy/2697049/ (accessed on 20 April 2022)). The initial set of amino acid sequences comprised a total of 295,199 entries for each protein.

For more targeted deciphering of genetic variation, we chose the spike RBD (amino acid sequence number 316–541) and created a dataset pairing the spike RBD with the M protein, ensuring they corresponded to the same strain. Initial pairing of sequences was performed using an in-house Python script, followed by multiple sequence alignment with MEGA X v10.2.6 [65] with the complete amino acid sequence of the SARS-CoV-2 Wuhan-Hu-1 strain (accession NC_045512, version NC_045512.2) serving as the reference sequence. Sequences with missing amino acids exceeding half of their total length were excluded, and duplicate sequences were represented by a single random instance from this study, also using the in-house Python script. After this filtration process, we obtained a final set of 39,847 sequences, with all spike RBDs and M proteins having sequence lengths standardized to 223 and 222 amino acids, respectively.

### 4.2. Mutational Synergy Analysis

#### 4.2.1. Single Mutations

First, we calculated the distributions of amino acids at each position in the sequence and the probability of mutation (PM (aa, x)) using Python, where aa denotes the specific amino acid, and x signifies the position within the sequence.

(1) $$\;PM\left(aa,\;x\right)=\frac{\mathrm{number}\;\mathrm{of}\;\mathrm{amino}\;\mathrm{acid}\;\mathrm{at}\;\mathrm{site}\;x}{\mathrm{total}\;\mathrm{number}\;\mathrm{of}\;\mathrm{sequences}}$$

Then, combining the mutations of interest reported by the WHO, we focused on the mutations that occurred for the first time in the Omicron variant and calculated their mutation rates.

#### 4.2.2. Multiple Mutations

In the spike RBD, the amino acid mutations were increased significantly. To ascertain the residue dependencies between different sites, we used an unsupervised statistical method known as EVmutation, which is adept at analyzing the interactions between protein variants and can explicitly account for interactions between various positions. The EVmutation process involves the following steps: (1) generation of multiple sequence alignments; (2) sample reweighting; (3) regularization; (4) calculation of the mutation effects.

To minimize potential interference, three independent experiments were conducted. The top 30 results were selected based on the score of each experiment and finally, 20 groups were chosen based on the highest frequency of occurrence in the overall results.

### 4.3. Sequence Translation

#### 4.3.1. Sequence-to-Sequence Transformer Model

**Figure 8:**
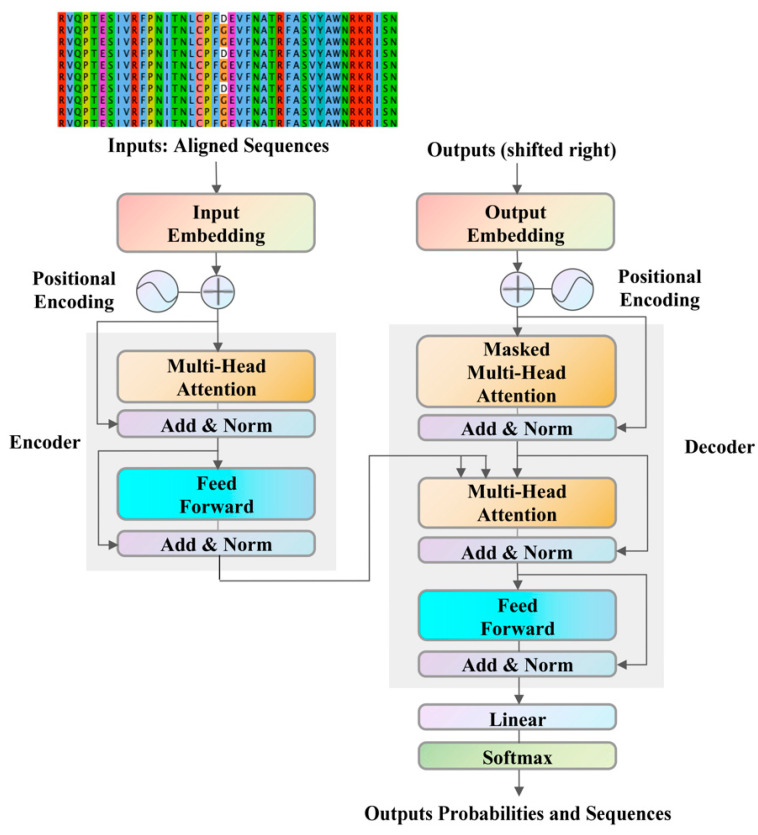
The Sequence-to-Sequence Transformer Model (S2STM) framework to realize mutual mapping between the RBD and M amino acid sequences.

We chose the Sequence-to-Sequence Transformer Model (S2STM) [54] to realize mutual mapping between the amino acid sequences of the spike RBD and M proteins (Figure 8). The model is structured as an encoder–decoder, with both the encoder and decoder comprising a stack of N_layers = 4. Within each encoder layer, there are two sublayers: a multi-head attention mechanism and a fully connected feed-forward network. Each decoder layer has three sublayers: a masked multi-head attention mechanism, a multi-head attention mechanism, and a fully connected feed-forward network.

The S2STM is based on the multi-head attention mechanism [66], which was developed from “Scaled Dot-Product Attention”. This mechanism is capable of refining feature information from multiple dimensions and effectively guards against overfitting. This single “Scaled Dot-Product Attention” function can be described as mapping a query and a set of key–value pairs to an output. In the calculation process, it is necessary to pack together into a matrix *Q*, *K*, and *V*; the dimensions are $d_{Q}$, $d_{k}$, and $d_{v}$, respectively. The function is defined as follows.

(2) $$Attention\left(Q,K,V\right)=softmax\;\left(\frac{QK^{T}}{\sqrt{d_{k}}}\right)V$$

Multi-head attention can group each single attention operation and it allows the model to jointly attend to information from different representation subspaces at different positions. The output is a linear transformation via learnable parameters, $W^{O}$. The multi-head attention function with n heads is articulated as follows.

(3) $$MultiHead\;\left(Q,K,V\right)\;=\;Contact\;\left({head}_{1},\ldots ,{head}_{n}\right)W^{O}\;\;\;$$

(4) $$where\;head_{i}=Attention\;\left(QW_{i}^{Q},KW_{i}^{K},VW_{i}^{V}\right)\;\;\;\;\;$$

#### 4.3.2. Model Parameters

All networks were simultaneously trained with a batch size of 8 on an NVIDIA 1080 GPU, adhering to computational resource efficiency and facilitating residual connections within the model. The values of input and output dimensionality were set as *d_model_* = 128, while the inner layer had a dimensionality of *d_ff_* = 512. To prevent overfitting, the number of parallel attention layers or heads was set as N_heads = 8. Consequently, for each head, the dimensions of $d_{k}=d_{v}=d_{model}/h$ = 16. In the training process, we utilized the Adam optimizer [67,68] with the following parameter settings: *β*_1_ = 0.9, *β*_2_ = 0.98, and *ε* = 10^−9^. Additionally, a dropout rate of *P_drop_* = 0.1 was employed as a generic parameter.

#### 4.3.3. Dataset Selection and Division

Before constructing the training datasets, it was necessary to create word sets tailored for the S2STM. This process enhanced the suitability of the datasets for the S2STM. Each amino acid position in the original sequence was expanded from one to three units, whereby each spike RBD sequence was described as a list of 223 3-g, and each M sequence was described as 222 3-g. The sizes of the spike RBD and M protein word sets were 1298 and 1144, respectively. These word sets were converted to numerical representations to be used as the index of the embedding.

The details of the training datasets are as follows: (1) The training datasets of the M sequences were divided equally into two parts: one was extracted from the whole M sequence datasets with mutations at sites 3/19/63, and the other was randomly extracted from the remaining sequences with an equal number of sequences, and (2) the training datasets of the spike RBD sequences introduced a bijection into the training datasets of the M sequences based on the isolate. The entire training and testing processes were performed using TensorFlow v2.0.4 [69]. We disrupted the datasets before training, and the datasets were divided into training, validation, and testing sets at a ratio of 0.8:0.1:0.1.

In parallel, we found that the core co-mutations in the spike RBD were G339D-S373P-S375F, S371F/L-S373P-S375F, and Q493R-Q498R-Y505H. To further investigate the potential covariation tendencies, we created six additional revised spike RBD testing datasets, named “G339-S373-S375”, “339D-373P-375F”, “S371-S373-S375”, “Q493-Q498-Y505”, and “493R-498R-505H”. For instance, “G339-S373-S375” (“339D-373P-375F”) indicated modifications of the 339/373/375 site into G, S, and S (D, P, and F), respectively. The remaining testing datasets were constructed following the same pattern.

#### 4.3.4. Model Validation

The calculations of the Pearson correlation coefficient (PCC) [55], Hamming distance [56], and Area Under Curve (AUC) [57] were conducted to assess the performance of the model.

The PCC serves as a metric for quantifying linear correlation between two datasets. Given a pair of random variables $\left(X,Y\right)$, the formula for ρ is

(5) $$\;\rho \;\left(X,Y\right)=\frac{cov\;\left(X,Y\right)}{\sigma_{X}\sigma_{Y}}$$

The Hamming distance, commonly employed for assessing the dissimilarity between two strings, assigns a distance value of 0 to indicate exact similarity. For two strings, $u=\left(u_{1},\cdots ,\;u_{n}\right)$ and $v=\left(v_{1},\cdots ,\;v_{n}\right)$, representing words in set C, the Hamming distance is denoted as *d(*u,v). Therefore, the Hamming distance functions as a metric within the set C.

The AUC serves as a metric for evaluating the discriminatory capability of a binary classifier and is utilized as a concise representation of the Receiver Operating Characteristic (ROC) Curve. A higher AUC value indicates superior performance of the model in discriminating between positive and negative classes.

(6) $$\mathrm{AUC}=\int_{0}^{1}\frac{\mathrm{True}\;\mathrm{Positive}}{\mathrm{True}\;\mathrm{Positive}+\mathrm{False}\;\mathrm{Negative}}\;d\left(x\right)\;\;$$

## 5. Conclusions

Our research identified some co-mutations in the spike RBD sequences during the evolution of SARS-CoV-2. Through a preliminary analysis of the sequences, we pinpointed the core mutations and several co-mutation sites in the spike RBD (G339D-S373P-S375F, S371F/L-S373P-S375F, and Q493R-Q498R-Y505H). Subsequently, we established, for the first time, mapping relationships between the RBD and M sequences using the S2STM. This model can reveal and validate the potential variation tendencies of the amino acid sequences of the spike RBD and M proteins with 99% accuracy. Our findings suggest that the co-mutation G339D-S373P-S375F is highly likely to induce mutations at site 3 in the M sequences, while the co-mutations Q493R-Q498R-Y505H are likely to induce mutations at sites 19 and 63 in the M sequences.

In our study, we propose that co-mutations in the spike RBD could potentially induce mutations in the M protein. This not only facilitates our understanding of the evolution of SARS-CoV-2 but also provides new insights into the mutational synergy between the spike RBD and M proteins, while simultaneously advancing the development of sequence analysis methodologies.

## Figures and Tables

**Table 1:** SARS-CoV-2 variants of concern and related spike RBD mutations and M mutations.

WHO Nomenclature or Designation	Pango Lineage	Spike RBD Mutations of Interest	M Mutations of Interest
Alpha	B.1.1.7	N501Y	
Beta	B.1351	K417N, N501Y, E484K	
Gamma	P.1	K417T, N501Y, E484K	
Delta	B.1.617.2	L452R, T478K	I82T
Omicron	B.1.1.529	**G339D**, **S371F/L**, **S373P**, **S375F**, **T376A**, **D405N, R408S**, K417N, **N440K**, **S447N**, T478K, E484A, **Q493R**, **Q498R**, N501Y, **Y505H**	**D3G, Q19E, A63T**, I82T

The bolded mutations occurred for the first time in the Omicron variant.

## Data Availability

Computational instructions and data of this work have been given in main text and supporting information; further information and requests may be directed and will be fulfilled by Zhiwei Yang (yzws-123@xjtu.edu.cn), the lead contact.

## Supplementary Materials

The following supporting information can be downloaded at https://www.mdpi.com/article/10.3390/ijms25094662/s1.
